# Ultra-processed food targets bone quality via endochondral ossification

**DOI:** 10.1038/s41413-020-00127-9

**Published:** 2021-02-26

**Authors:** Janna Zaretsky, Shelley Griess-Fishheimer, Adi Carmi, Tamara Travinsky Shmul, Lior Ofer, Tali Sinai, Svetlana Penn, Ron Shahar, Efrat Monsonego-Ornan

**Affiliations:** 1 School of Nutrition Science, Institute of Biochemistry, Food Science and Nutrition, The Hebrew University, Rehovot, 76100 Israel; 2 Koret School of Veterinary Medicine, The Robert H. Smith Faculty of Agriculture, Food, and Environment, The Hebrew University, Rehovot, 76100 Israel

**Keywords:** Metabolic bone disease, Bone

## Abstract

Ultra-processed foods have known negative implications for health; however, their effect on skeletal development has never been explored. Here, we show that young rats fed ultra-processed food rich in fat and sugar suffer from growth retardation due to lesions in their tibial growth plates. The bone mineral density decreases significantly, and the structural parameters of the bone deteriorate, presenting a sieve-like appearance in the cortices and poor trabecular parameters in long bones and vertebrae. This results in inferior mechanical performance of the entire bone with a high fracture risk. RNA sequence analysis of the growth plates demonstrated an imbalance in extracellular matrix formation and degradation and impairment of proliferation, differentiation and mineralization processes. Our findings highlight, for the first time, the severe impact of consuming ultra-processed foods on the growing skeleton. This pathology extends far beyond that explained by the known metabolic effects, highlighting bone as a new target for studies of modern diets.

## Introduction

The vertebrate skeleton has evolved as a dynamic system that serves numerous functions, such as protecting internal organs, creating attachment sites for muscles to produce locomotion, providing a reservoir for minerals, and serving as a hematopoietic niche. Many signaling pathways control the patterning, growth and maturation of skeletal structures from early development to adulthood.^1^

The vertebrate bony skeleton is formed through two processes, intramembranous ossification or endochondral ossification (EO),^1,2^ the latter of which is responsible for long bone development. EO is initiated in the fetus and continues postnatally in the growth plates (GP) until growth cessation in adolescence.^2,3^ The rate of longitudinal bone growth is determined by the rate of chondrocyte proliferation, the size of the hypertrophic chondrocytes, and cartilage degradation and replacement by bone. Chondrocytes in different zones of the GP are distinguished by their differentiation stage, morphology and matrix production. In the proliferative zone (PZ), chondrocytes are arranged in longitudinal columns, where they rapidly proliferate and deposit typical cartilage extracellular matrix (ECM) components, especially collagen type 2 (Col2) and aggrecan (Acan). Next, chondrocytes exit the cell cycle and become prehypertrophic by expressing the type X collagen (*Col10*) and Indian hedgehog (*Ihh*) genes. Prehypertrophic chondrocytes increase in size to become hypertrophic cells.^1,4^ In the hypertrophic zone (HZ), cells become enlarged, swollen and vacuolated and are characterized by secretion of *Col10*, alkaline phosphatase, and angiogenic factors.^5^ Fully differentiated hypertrophic chondrocytes at the chondro-osseous junction undergo apoptosis or transdifferentiation, and cartilaginous ECM is removed by osteoclasts, allowing the penetration of blood vessels and bone tissue formation by osteoblasts.^1,2,4,6^ The EO process is tightly orchestrated by various signaling molecules and transcription factors, including transforming growth factor β/bone morphogenetic protein (BMP), fibroblast growth factor (FGF), Wnt, hedgehog, and the transcription factor Sox9.^1,2^

Bone elongation and hence whole-body longitudinal growth are strictly regulated by genetic and environmental elements. To maximize growth and peak bone mass, modifiable environmental factors, particularly the balance of nutrition, should be optimized before the onset of puberty and maintained throughout this period of rapid growth.^7^ Proper intake of macronutrients, minerals and vitamins is therefore of great importance in achieving optimal bone structure and quality.^8^

Food supplies in recent decades have been dominated by heavily processed, ready-to-eat products. Essentially, 75% of all world food sales are of processed foods.^9,10^ Over the past 30 years, children’s ultra-processed food (UPF) intake has increased markedly, with 50% of the children in the US consuming these foods.^11^ Only in the US does UPF comprise 57.9% of energy intake, of which 89.7% is derived from added sugars. This reflects children’s excessive consumption of food and drink that are high in fat and refined sugars but do not provide appropriate levels of the proteins, vitamins and minerals required for growth.^12^ The health outcomes resulting from this type of nutrition have been described in many studies and include obesity, metabolic syndrome and diabetes.^13,14^ However, the critical effect of such a diet on postnatal skeletal growth from birth until puberty has not been assessed.^12^

While previous studies have shown that malnutrition or malabsorption result in longitudinal growth retardation and short stature,^15^ the effect of excessive consumption of UPF on bone development and growth has not been evaluated at the cellular level, nor has its effect on bone quality been studied. Here, we used young rats as our experimental model for postnatal development and examined the effect of an unbalanced ultra-processed diet (UPD) during postnatal growth on skeletal development and quality.

## Results

### Effect of UPD on growth and physiological parameters

To study the link between UPF consumption and postnatal skeletal development, we conducted a 6-week-long in vivo trial in young female rats (3 weeks to 9 weeks of age). This time frame extends from weaning to puberty and therefore represents the growth period before sexual maturation and GP closure in humans.^16^

The diet chosen here exemplifies the Western UPD with unbalanced levels of micro- and macronutrients.^12^ The experimental design included two groups: the control group, which was fed a standard rat diet, and the UPF + CSD group, which was fed a diet comprised of a typical UPF meal (see methods for preparation) and a caloric soft drink. All rats had *ad libitum* access to food and liquids.

**Fig. 1:**
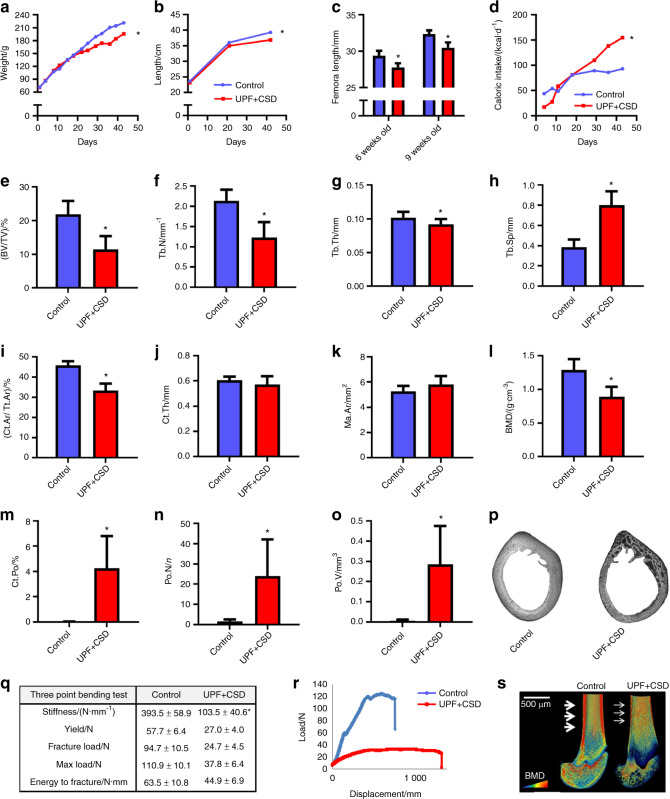
Consumption of an ultra-processed diet leads to growth retardation and to alterations in bone architecture and biomechanical properties. The control group, which received a standard diet for growing rats, was compared to the UPF + CSD group that received a diet based on UPF and a caloric soft drink. **a** Body weight. **b** Total length from nose to tail. **c** Femur length at 6 and 9 weeks of age was measured using SkyScan software. **d** Daily caloric intake (kcal·d^−1^ per rat). **e**–**o** Femur µCT analyses at 9 weeks of age. **e**–**h** Trabecular parameters: bone volume fraction (BV/TV), trabecular number (Tb. N), trabecular thickness (Tb. Th), and trabecular separation (Tb.Sp). **i**–**l** Cortical parameters: cortical area fraction (Ct.Ar/Tt. Ar), average cortical thickness (Ct. Th), medullary area (Ma. Ar) and bone mineral density (BMD). **m**–**o** Bone porosity parameters: cortical porosity (Ct. Po), pore number (Po. N) and total pore volume (Po. V). **p** Light microscopy images of cross-sections of rat cortical bone representing cortical bone porosity. **q** Biomechanical properties: stiffness (N·mm^–1^), yield (N), fracture load (N), max load (N) and energy to fracture (N·mm), assessed by three-point bending test. **r** Representative three-point bending load-displacement curves of the control and UPF + CSD bones. **s** Representative 3D images of cross-sections of femur bone visualized by Amira software, with arrows indicating cortical thickness. Values are expressed as the mean ± SD, *n* = 8. *P < 0.05 compared to control

During the experiment, the body weight and total body length of all rats were measured. In addition, the femur and lumbar vertebra lengths were assessed by µCT after 3 and 6 weeks of the experiment. Weight gain was reduced in the UPF + CSD group (Fig. 1a), and total body and femoral lengths were significantly shorter in this group than in the control group (Fig. 1b, c). Surprisingly, although growth in the UPF + CSD group was retarded, caloric intake (derived from both food and the caloric soft drink) was significantly higher (Fig. 1d). These results demonstrated that UPD consumption stunts growth, but this is not due to caloric deficiency.

### Effect of the UPD on bone quality

To assess the effect of the UPD on bone quality, femora and lumbar vertebrae were scanned by µCT to examine their trabecular and cortical bone properties.

The trabecular bone parameters of the UPF + CSD group were inferior to those of the controls. The bone volume fraction (BV/TV) of the femora decreased significantly, from 35.54% to 19.17% and from 23.06% to 16.27% at 6 and 9 weeks of age, respectively. The mean trabecular number (Tb. N) and mean trabecular thickness (Tb. Th) in the femora were significantly lower in the UPF + CSD group, while trabecular separation (Tb. Sp), which represents the mean distance between trabeculae, was significantly higher than that in the control group at both time points (Fig. 1e–h and Supplementary Table 1). Analysis of the vertebrae showed very similar patterns based on the trabecular analyses of the UPF + CSD vs. control groups (Supplementary Table 1).

Furthermore, cortical bone analysis of the UPF + CSD group demonstrated severe deterioration compared to rats fed the control diet. The deleterious effects included a decline in the cortical area fraction (Ct.Ar/Tt. Ar) and BMD, which was 30% lower for rats consuming UPF + CSD (Fig. 1i, l and Supplementary Table 1). Ct. Po was significantly higher in the UPF + CSD group. The number of pores (Po. N) was 15 times higher in the UPF + CSD group, and the total volume of pores (Po. V) increased 47-fold (Fig. 1m–o, Supplementary Table 1). Typical cross sections from the two groups revealed the sieve-like appearance of the cortical bone in the UPF + CSD group compared to the normal-looking cortices in the control group (Fig. 1p).

The three structural parameters that have the largest effect on the mechanical performance of bones are the geometry, mineral density and porosity of the cortical bone. Our results led us to expect the deterioration of the mechanical stiffness and strength of the femora, and a three-point bending test of the femora was therefore conducted.^17^ A significant and dramatic decrease was demonstrated in bone mechanical parameters such as stiffness, yield load, maximum load and fracture load (Fig. 1q, r, Supplementary Table 1).

Taken together, these results demonstrated the deterioration in the structural and mechanical features of bones from the UPF + CSD vs. Control group, as seen in the Amira image (Fig. 1s). The deterioration in these properties was observed as early as 6 weeks of age (Supplementary Table 1).

### Effect of UPF on GP organization

**Fig. 2:**
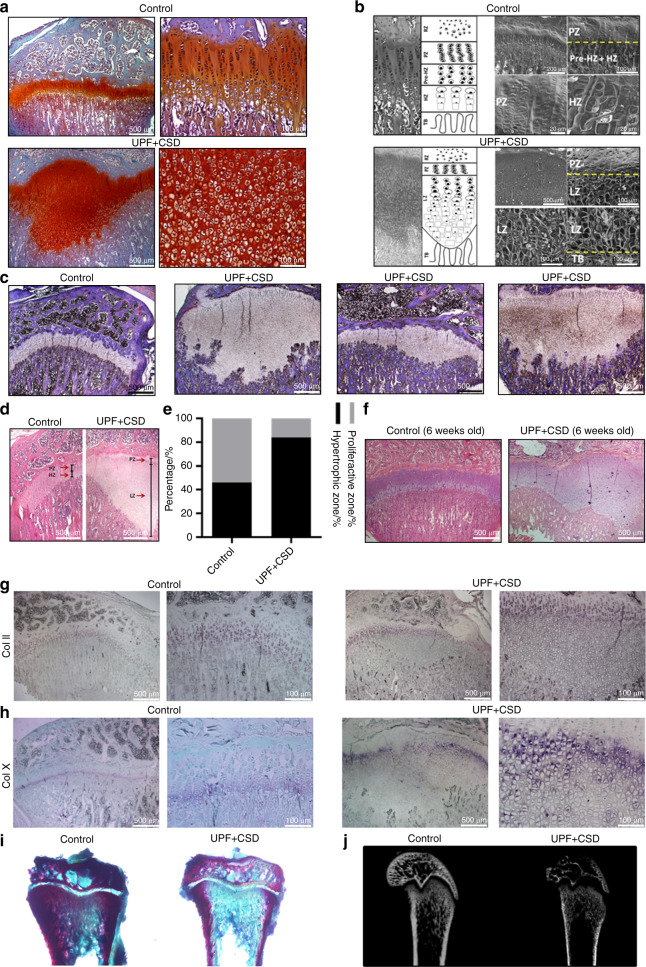
Consumption of an ultra-processed diet results in a damaged growth plate (GP) and modified chondrocyte differentiation process. Tibiae from the control and UPF + CSD groups (see legend to Fig. 1) were dissected, processed, embedded in paraffin blocks and stained with (**a**) safranin O, (**c**) Masson’s trichrome or (**d** and **f**) hematoxylin and eosin. **b** Light microscopy and schematic presentation (left) and surface scanning by electron microscopy (right) of the different GP zones. Resting zone (RZ), proliferative zone (PZ), prehypertrophic zone (Pre-HZ), hypertrophic zone (HZ), lesion zone (LZ), trabeculae (TB). **c** Masson’s trichrome staining demonstrates a variety of GP lesions. **d** Hematoxylin and eosin staining of the control and UPF + CSD groups. The different zones are depicted by arrows. **e** Quantification of the relative ratio of the zones in the GP: the widths of the whole GP and the PZ, HZ and LZ were measured at 10 different points along the GP and averaged with measurements from 6 other GP samples in each group. The percentages of PZ and HZ/LZ from the whole GP were calculated. **f** GP lesion at 6 weeks of age. **g**, **h** In situ hybridization analysis of Col II (top) and Col X (bottom) mRNA signals. **i** Alizarin red and alcian blue staining of nondecalcified tibial bone. **j** 2D X-ray image of femora bone

Abnormal bone phenotypes may stem from interference with the EO process in the GP^2^. We therefore performed a histological analysis of the GP in 6-week- and 9-week-old rats. Sections stained with safranin O (which distinguishes cartilage from bone) revealed an uneven widening of the GP in rats in the UPF + CSD group. This widening was characterized by a mass of avascular nonmineralized cartilage lesions that extended from the epiphyseal GP into the metaphysis of the tibia (Fig. 2a). The details of the organization of the UPF + CSD group LZ, in comparison to the typical organization of the GP, were visualized by SEM. The cells of the LZ lost their columnar orientation, were varied in size and were larger than proliferative cells but smaller than hypertrophic cells compared to the cells of the GP in the control group (Fig. 2b). Interestingly, the different bones demonstrated variability in terms of size, shape and localization within the GP (Fig. 2c). To quantify this unique GP appearance, we measured the length of each GP region (PZ, HZ and LZ) (Fig. 2d). Enlargement of the GP and the different proportions of the proliferative vs. nonproliferative regions in the GP were observed in the UPF + CSD rats (Fig. 2e). These results suggested that the harmful effect of the UPD on bone growth and quality occurs initially through disruption of the normal EO process in the GP at as early as 6 weeks of age (Fig. 2f). In addition, in situ hybridization of the two main matrix collagens revealed a strong signal for *Col2* mRNA in the PZ and a *Col10* signal in the hypertrophic chondrocytes above the cartilaginous plaque. The absence of *Col10* expression in the cartilage extension suggests that the cells in the LZ have lost their hypertrophic properties (Fig. 2g, h). Moreover, the bones in the UPF group exhibited decreased mineralization, as the cartilaginous plaque in the UPF group was not calcified (Fig. 2i, j).

### Effect of UPF on the GP transcriptome

**Fig. 3:**
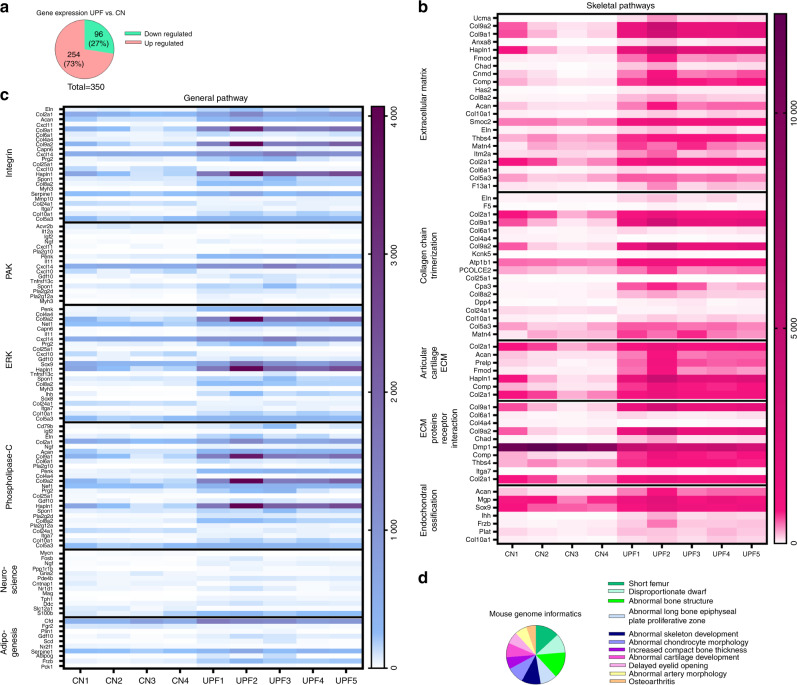
Consumption of an ultra-processed diet modifies the growth plate (GP) transcriptome. **a** Pie chart representing 302 upregulated (pink) and downregulated (green) genes [ultra-processed food (UPF) vs. control (CN) groups]. **b** Heatmap representing the expression signature of differentially expressed genes clustered according to different skeletal pathways. **c** Heatmap representing the expression signature of differentially expressed genes clustered according to general pathways. **d** Pie chart representing the frequency of differentially expressed genes in the mouse genome using MGI phenotype analysis. For detailed gene expression, see Supplementary Table 2

To elucidate and characterize the disrupted GP phenotype, we performed mRNA sequencing. For mRNA library preparation, we isolated only the tibial GPs from the control and UPF groups. Four control and five UPF mRNA libraries were produced and sequenced. Out of 12 848 genes, 302 were differentially expressed. Of the 302 genes, 74% were upregulated in the UPF group, and 125 were related to the EO process, which revealed a gene profile that is typical for the chondrocyte differentiation pathway, matrix production and matrix calcification. GeneAnalytic software was used to reveal the possible biological patterns, canonical pathways and networks in the GP (Fig. 3a–c and Supplementary Table 2). MGI phenotype analysis, which determines the outcome of either naturally occurring or induced mouse mutations,^18^ reveals the correlations of phenotypes with different skeletal diseases and abnormalities, such as short femurs, dwarfism and other conditions (Fig. 3d). Taken together, these findings suggested that GP cartilage cells more actively produced ECM components, which may have led to impairment of the EO process.

### ECM formation and degradation

**Fig. 4:**
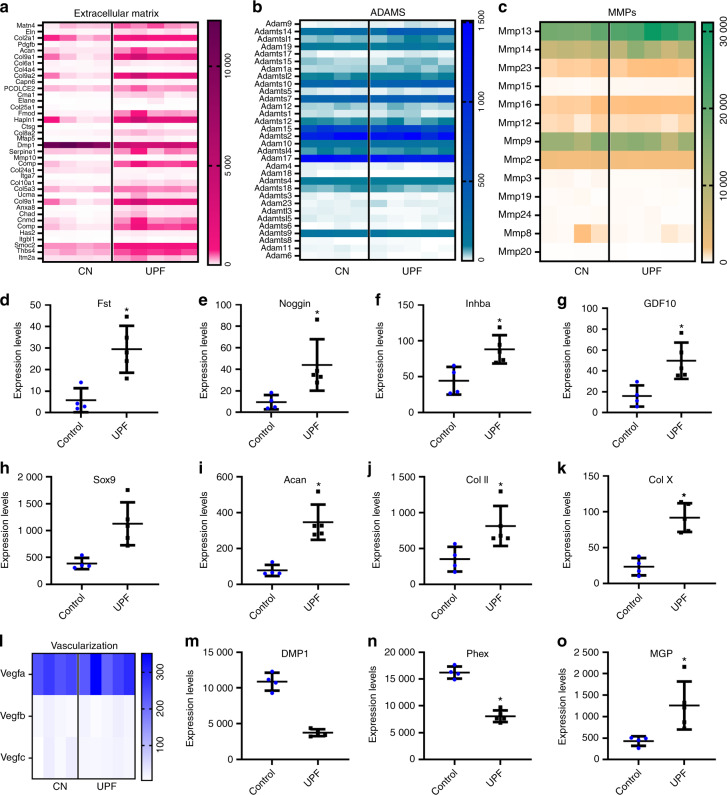
Consumption of an ultra-processed diet affects extracellular matrix (ECM) formation and degradation. Heatmaps representing the expression signature of differentially expressed genes in the (**a**) ECM pathway, (**b**) A disintegrin and metalloproteinase (ADAM) gene family, and (**c**) matrix metalloproteinase (MMP) gene family. **d**–**k** Expression frequency of genes associated with growth plate (GP) chondrocyte proliferation and differentiation. BPM signaling pathway: **d** Fst, **e** Nog, **f** Inhba and **g** Gdf10; Sox signaling pathway: **h** Sox9, **i** Acan, **j** Col II and **k** Col X. **l** Heatmap representing the expression signature of the VEGF gene family. Expression frequency of genes associated with the mineralization process: **m** DMP1, **n** Phex, **o** MGP. The uniquely mapped reads per gene were counted using HTSeq-count, and differential expression analysis was performed using the DESeq2 R package. * Denotes significant differences from the control group. Genes were considered significantly expressed if the adjusted *P* value was lower than 0.05. Groups: Control (*n* = 4) and ultra-processed food (UPF) (*n* = 5)

We found significant upregulation in the production of ECM components in the UPF group, including 38 genes encoding matrix proteins such as cartilage oligomeric matrix protein (COMP), *Acan*, and *Col2*, -*4*, -*9* and -*10* (Fig. 4a). Intriguingly, the elevation in ECM components was not accompanied by a corresponding elevation in matrix-degrading enzymes, matrix metalloproteinases (MMP), or A disintegrin and metalloproteinase (ADAM), which showed similar levels of expression in both groups (Fig. 4b, c). These findings suggest an alteration in the balance between matrix formation and degradation.

### Proliferation and differentiation processes

The UPF group exhibited increased expression of genes encoding the BMP inhibitors follistatin (*Fst*), noggin (*Nog*), *Gdf10* and inhibin beta A subunit (*Inhba*),^4^ suggesting the attenuation of BMP signaling (Fig. 4d–g). Moreover, the expression of the transcription factor sex-determining region Y (SRY)-box 9 (*Sox9*) was upregulated in the UPF group in tandem with a significant increase in the expression of the downstream genes *Gdf10*, *Acan*, *Col2* and *Col10* (Fig. 4h–k). BMP and *Sox9* are key regulators of GP chondrocyte proliferation and differentiation, implying the impairment of these processes. Taken together, these results suggest that an imbalance in proliferation and differentiation results in a disorganized GP structure.

### GP mineralization and vascularization

Consistent with the enlarged GP, the mineralization process was altered in the UPF group by the downregulation of dentin matrix acidic phosphoprotein 1 (*Dmp1*) and phosphate-regulating endopeptidase homolog X-linked (*Phex*), encoding proteins that enhance mineralization,^22,23^ and upregulation of the gene encoding matrix gla protein (MGP), a mineralization inhibitor.^24^ Interestingly, while mineralization was downregulated, vascularization genes (*vegfa*, *vegfb*, *vegfc* and *PECAM-1*) were not differentially expressed (Fig. 4l–o). This suggested that mineralization is not altered due to disruption of vascularization. Liu et al.^19^ showed that *Dmp1* or *Phex* knockout leads to the elevation of *FGF23* and, as a result, to hypophosphatemic rickets.^19^ This was not the case in our model, where serum analysis showed reduced levels of FGF23, hyperphosphatemia and hypocalcemia followed by high levels of PTH and low levels of osteoprotegerin (OPG), indicating that bone resorption was taking place (Supplementary Table 3). Correspondingly, cortical TRAP staining demonstrated higher osteoclast activity together with increased porosity in the UPF group (Supplementary Fig. 1).

In summary, the GP transcriptome of the UPF group demonstrated what appeared to be a disrupted EO with alterations in ECM formation and degradation and disruption in proliferation–differentiation mechanisms and mineralization.

### Effect of the macro- and micronutrient composition on bone growth and phenotype

To determine the main cause for the observed phenotype characterized by growth retardation, bone quality deterioration and an altered mRNA profile, a series of animal trials was conducted (Supplementary Fig. 2).

The soft drink used in the study contained high levels of phosphoric acid, which might be associated with reduced accrual of bone minerals.^20,21^ Therefore, we further studied the specific effect of this soft drink on metabolic and bone parameters. Rats received the balanced control diet with a caloric soft drink or noncaloric soft drink. No dramatic differences were found between the rats consuming a normal diet with caloric and noncaloric soft drinks and the control group with respect to metabolic parameters, bone quality, structure or growth (Supplementary Fig 3 and Supplementary Text). Furthermore, rats that consumed UPDs with or without a soft drink beverage exhibited the same disrupted phenotype with or without the soft drink (Supplementary Fig. 4 and Supplementary Text). Hence, we concluded that the harmful effects were not caused solely by the soft drink.

**Fig. 5:**
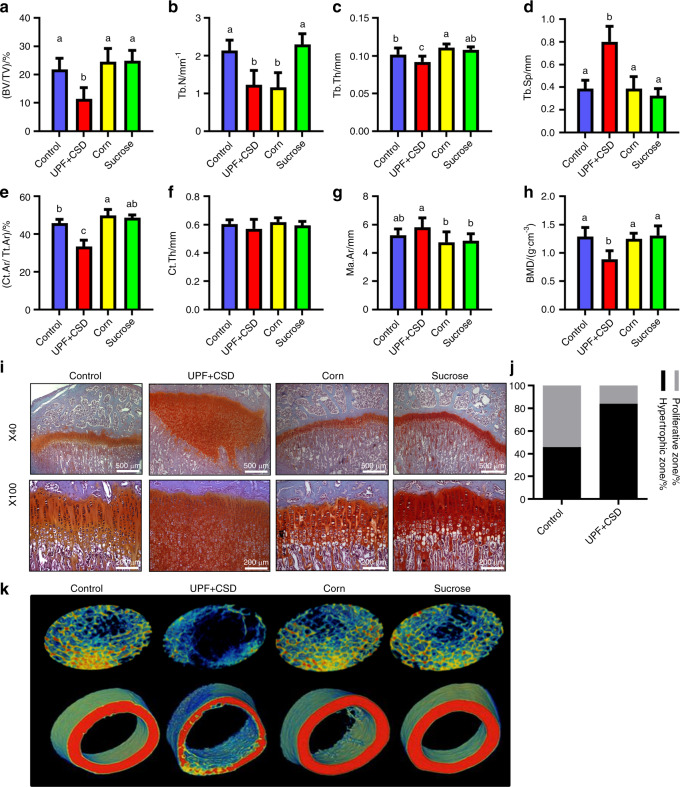
Ultra-processed food (UPF) macronutrients (fats and sugar) are not responsible for alterations in bone architecture and biomechanical properties. The control and UPF groups were compared to a group receiving a high-fat diet based on the addition of corn oil (Corn) and a group fed a high-sucrose diet including a control diet + drinking solution with 10% sucrose (sucrose). **a**–**h** Femur µCT analyses. **a**–**d** Trabecular parameters: bone volume fraction (BV/TV), trabecular number (Tb. N), trabecular thickness (Tb. Th), and trabecular separation (Tb.Sp). **e**–**h** Cortical bone parameters: cortical area fraction (Ct.Ar/Tt. Ar), average cortical thickness (Ct. Th), medullary area (Ma. Ar) and bone mineral density (BMD). **i** Growth plates (GPs) from the control, UPF + CSD, corn and sucrose groups were stained with safranin-O. **j** Quantification of the relative ratio of the zones in the GP. **k** Representative 3D images of transverse sections of femoral trabecular and cortical areas visualized by Amira software. Values are expressed as the mean ± SD, *n* = 8. Different letters denote significant differences at *P* < 0.05 between groups

To test whether one of the macronutrients that characterizes the UPD is responsible for the bone phenotype, we conducted an experiment with diets that isolated the main ingredients of the UPD: fats and sugar. One group of rats received a high-fat diet based on the addition of corn oil (Corn), and the second group was fed a high-sucrose diet that consisted of the control diet + drinking solution with 10% sucrose (sucrose). The results showed normal trabecular and cortical bone parameters and normal GP organization in the Corn and Sucrose groups (Fig. 5, Supplementary Table 1). Thus, the macronutrients did not cause the phenotype.

**Fig. 6:**
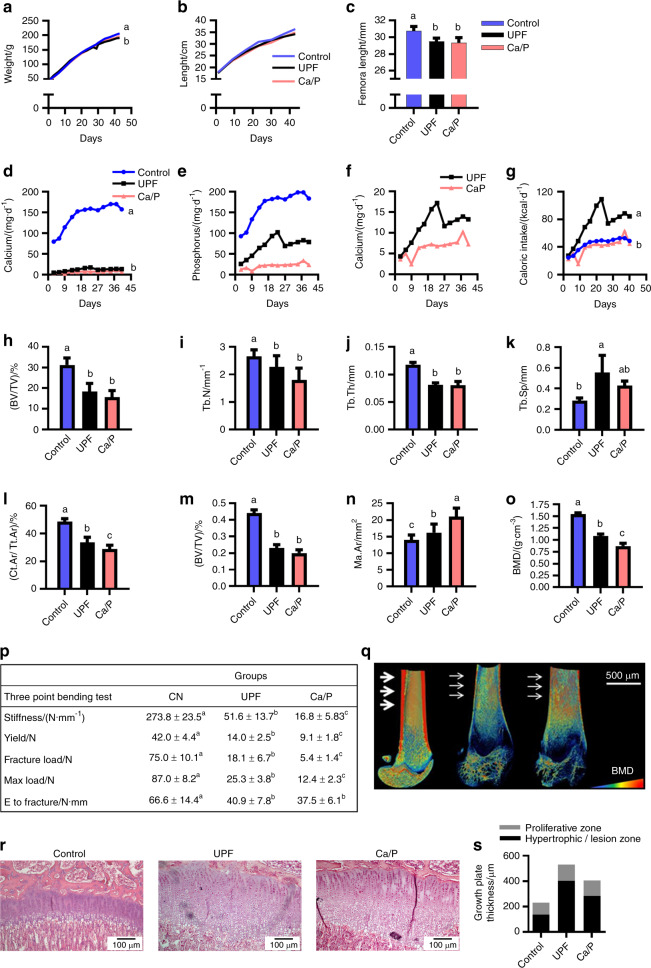
Ca/P deficiency is partially responsible for the altered bone phenotype following ultra-processed food (UPF) consumption. Control and UPF groups were compared to a group receiving a diet that mimicked the Ca (0.62 mg·g^−1^) and P (1.21 mg·g^−1^) levels and ratio in the ultra-processed diet (Ca/P group). **a** Body weight. **b** Total length from nose to tail. **c** Femur length at 9 weeks of age. **d** Daily caloric intake (kcal·d^−1^ per rat). **e** Daily P intake (mg·d^−1^ per rat). **f** Daily Ca intake by the UPF and Ca/P groups vs. the control group (mg·d^−1^ per rat). **g** Daily Ca intake by UPF vs. Ca/P group (mg/rat per day). **h**–**o** Femur µCT analyses. **h**–**k** Trabecular parameters: bone volume fraction (BV/TV), trabecular number (Tb. N), trabecular thickness (Tb. Th), and trabecular separation (Tb.Sp). **l**–**o** Cortical bone parameters: cortical area fraction (Ct.Ar/Tt. Ar), average cortical thickness (Ct. Th), medullary area (Ma. Ar) and bone mineral density (BMD). **p** Representative 3D images of femur bones visualized by Amira software. **q** Biomechanical properties: stiffness (N·mm^−1^), yield (N), fracture load (N), max load (N) and energy to fracture (N·mm), assessed by three-point bending test; CN, control. **r** Growth plates (GPs) from the control, UPF and Ca/P groups were stained with hematoxylin and eosin. **s** Quantification of the relative ratio of the zones in the GP. Values are expressed as the mean ± SD, *n* = 8. Different letters denote significant differences at *P* < 0.05 between groups

Next, we analyzed the micronutrient composition of the diets and found low micronutrient contents (Ca, P, zinc, iron, magnesium and copper) in the UPD (Supplementary Fig. 5). However, since rats in the UPF group ate significantly more food, they obtained the appropriate amounts of all micronutrients except for Ca and P (data not shown). Thus, we conducted the next trial using a custom diet that mimicked the Ca (0.62 mg·g^−1^) and P (1.21 mg·g^−1^) levels and ratio in the UPD (Ca/P group). The Ca/P group exhibited growth retardation in terms of body weight, body length and femoral length that similar to the UPF group (Fig. 6a–c). Interestingly, for the duration of the experiment, the Ca/P group consumed even less Ca and P than the UPF group (Fig. 6d–g); as a result, their PTH and FGF23 serum levels were upregulated, as was observed in the UPF group (Supplementary Table 3C). Nevertheless, the serum levels of Ca (8.1 ± 0.9 vs. 6.3 ± 0.8), phosphate (7.24 ± 1.4 vs. 8.9 ± 0.7) and alkaline phosphatase (272.9 ± 34.2 vs. 374.5 ± 50.8) were better balanced in the Ca/P vs. UPF group (Supplementary Table 3D). µCT analysis of the Ca/P group demonstrated similar or worse bone patterns than the UPF group (Fig. 6h–o). Figure 6p displays the Amira image of the three groups. Furthermore, the bone mechanical properties were significantly worsened in the Ca/P group; for instance, Ca/P femoral stiffness was 3 times lower than that in the UPF group (Fig. 6q). These results suggested that balanced blood values were achieved at the expense of bone.

Histological analysis showed milder lesions in 42% of the GPs in the Ca/P group compared to 86% of the GPs in the UPF group (Fig. 6r). Moreover, GP thickness measurements showed reduced GP extension in the Ca/P group compared to that in the UPF group (Fig. 6s).

Altogether, we demonstrated that the main cause of the phenotype is neither the caloric soft drink nor the unbalanced macronutrient levels. While there are several similarities in the phenotypes of the UPF and Ca/P groups, we cannot conclude that the same mechanisms are responsible for these results.

### Eating patterns

**Fig. 7:**
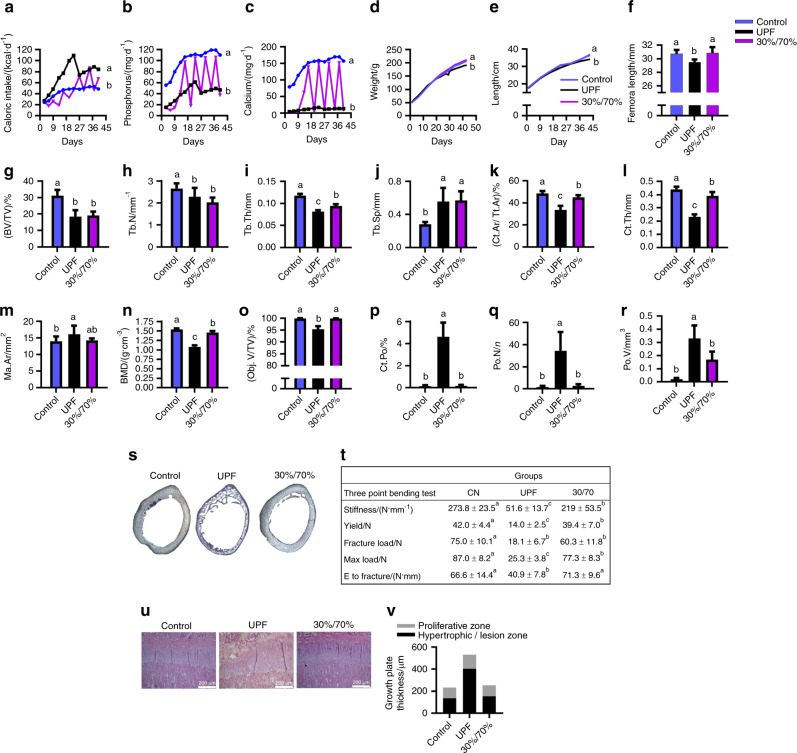
Partial consumption of ultra-processed food (UPF) leads to impaired skeletal quality. The UPF and control groups were compared to a group that received a balanced control diet 30% of the week and an ultra-processed diet (UPD) the rest of the week (30%/70% group). **a** Daily caloric intake (kcal·d^−1^ per rat). **b** Daily P intake (mg/rat per day). **c** Daily Ca intake (mg/rat per day). **d** Body weight. **e** Total length from nose to tail (cm). **f** Femur length (cm) at 9 weeks of age. **g**–**r** Femur µCT analyses. **g**–**j** Trabecular parameters: bone volume fraction (BV/TV), trabecular number (Tb. N), trabecular thickness (Tb. Th), and trabecular separation (Tb.Sp). **k**–**n** Cortical parameters: cortical area fraction (Ct.Ar/Tt. Ar), average cortical thickness (Ct. Th), medullary area (Ma. Ar) and bone mineral density (BMD). **o**–**r** Bone porosity parameters: percent object volume (Obj. V/TV), cortical porosity (Ct. Po), pore number (Po. N) and total pore volume (Po. V). **s** Light microscopy images of cross-sections of rat cortical bone representing cortical bone porosity. **t** Biomechanical parameters assessed by three-point bending test; CN, control. **u** Tibiae from the control, UPF and 30%/70% groups were dissected, processed and stained with hematoxylin and eosin. **v** Quantification of the relative ratio of the zones in the growth plate (GP). Values are expressed as the mean ± SD, *n* = 8. Different letters denote significant differences at *P* < 0.05 between groups

It was of interest to examine the eating patterns and frequency of UPF consumption in children compared to that in our preclinical model. To this end, we planned an experiment mimicking the actual UPF consumption rate among children. In this experiment, rats were provided with the balanced control diet 30% of the week and the UPD the rest of the week (30%/70% group). Despite the lower consumption of calories, Ca and P in the 30%/70% group (Fig. 7a–c), the growth patterns were similar to those in the control group (Fig. 7d–f). However, with respect to bone quality, except for the trabecular thickness, the trabecular bone parameter values were similar to those of the UPF group (Fig. 7g–j). The cortical parameters were improved but did not reach the control levels, while the cortical porosity was comparable to that in the control group (Fig. 7k–s). Moreover, the bone mechanical properties in the 30%/70% group, although improved, also did not reach the control levels, demonstrating reduced bone stiffness, yield, fracture load and maximal load (Fig. 7t). On the other hand, the GP organization was comparable with that in the control (Fig. 7u, v). These results proved that even partial consumption of a UPD leads to impaired skeletal quality.

## Discussion

Extensive consumption of processed food and soft drinks is endemic in the modern era, with many known metabolic implications.^11^ However, to the best of our knowledge, the impact of these food choices on skeletal development has not been studied.^25,26^

We showed that consumption of a diet consisting exclusively of UPF in growing rats leads to severe disruption of skeletal development, which is characterized by major alterations of the GP. We observed significant deterioration of the quality of trabecular and cortical bone, widely distributed porosities in the compact bone, and a dramatic decrease in the BMD, all of which were correlated with retardation of longitudinal growth and bone fragility.

The most striking result was the observation of aberrant cartilage occupying the GP of rats fed a UPD. Histological and SEM analyses of these bones revealed a disordered GP characterized by a noncalcified, nonvascularized cartilage lesion that extended from the epiphyseal GP into the metaphysis. This rare and severe phenotype differs greatly from other abnormal GP phenotypes, such as rickets and protein-energy malnutrition.^16^ To shed light on the underlying mechanism of the disorder of the GP, we performed mRNA-seq of control and UPD GP samples. The mRNA profile of the UPF group exhibited an impaired EO process.

The UPD caused a significant increase in the synthesis of ECM components, while there was no difference in the expression of matrix-degrading enzymes, leading to an imbalance in the ECM formation–degradation process. This might have led to the formation of cartilaginous plaques in the GP of the UPF group. BMP signaling was weakened due to high expression of BMP inhibitors (*Fst*, *Nog*, *Gdf10* and *Inhba*) without modifications of BMP or its receptors. BMP plays an essential role at every stage of endochondral bone development.^4^ It can induce bone and cartilage formation and stimulate chondrocyte proliferation and differentiation.^5,27,28^

Another fundamental factor for EO is *Sox9*, which is crucial for all phases of the development of the chondrocyte lineage from early condensation to conversion from proliferating to hypertrophic chondrocytes.^4^ *Sox9* is known to directly regulate *Col2a1*,^29^ *Acan*^30^ and *Gdf10* expression in the PZ.^31^ Our results demonstrated enhanced expression of *Sox9* mRNA, which could explain the upregulation of its downstream genes (*Col2a1*, *Acan* and *Gdf10*), suggesting the intensification of the early chondrocyte differentiation process.

In addition to *Sox9* elevation, the levels of *Col10*, a hypertrophic matrix component, were upregulated in the UPF group. He et al.^32^ confirmed the requirement of *Sox9* for hypertrophic chondrocyte development. They demonstrated that *Sox9* and *Jun* bind and coactivate a *Col10a1* enhancer. Furthermore, Dy et al.^33^ claimed that *Sox9* is necessary for GP chondrocyte hypertrophy. They showed that *Sox9* transactivates *Col10a1* along with *Mef2c* to increase hypertrophy and maintain a functional GP.^33^ We showed that while *Sox9* and *Col10* were upregulated in the UPF group, *Jun* and *Mef2c*, though highly expressed, were not differentially expressed. This might imply that there are more candidates responsible for the elevated Col X levels, such as *FoxA* factors. Indeed, *FoxA2* and *A3* were upregulated in the UPF group by 2.8- and 3.2-fold, respectively. *FoxA2* and *A3* regulate chondrocyte hypertrophy through direct induction of *Col10.*^27^ Accordingly, Ionescu et al.^28^ showed that chondrocyte hypertrophy is delayed in Col II–Cre, FoxA2^flox/flox^, and FoxA3^−/−^ mice, suggesting that these factors are responsible for the elevation of *Col10a1* in our model.

Our results also demonstrated downregulation of the crucial ossification genes *Dmp1* and *Phex* and upregulation of MGP. Dmp1 and Phex belong to a group of proteins that enhance mineralization,^22,23^ whereas MGP is a mineralization inhibitor,^24^ suggesting the inhibition of the bone mineralization process. Ye et al.^34^ showed that Dmp1^−/−^ mice have shorter bones and increased tibia width and cortical porosity. They further reported that Dmp1^−/−^ mice have a disorganized and irregular GP with an expanded HZ that is poorly calcified.^34^ Phex^−/−^ mice exhibited a very similar phenotype, including a widened and irregular HZ with hypomineralization and increases in cartilage remnants from the GP in both trabecular and cortical bone.^35^ These results correspond to the phenotype exhibited in the UPF group in our experiment.

Inactivation of either *Dmp1* or *Phex* leads to overexpression of *FGF23* by osteocytes and, as a result, to hypophosphatemic rickets.^19,34,36,37^ This contradicts our findings of a decrease in serum *FGF23* and hyperphosphatemia. These differences may result from the fact that knockout mice are characterized by complete deficiency in *Dmp1* and *Phex*, whereas our UPF group exhibited a decrease in *Dmp1* and *Phex* mRNA levels. Moreover, our experimental group also exhibited hypocalcemia and thus the elevation of serum *PTH*, leading to a decline in *OPG* levels. *OPG* is known as a receptor antagonist for *RANKL* that prevents it from binding to and activating *RANK*, thereby inhibiting osteoclast differentiation.^38^ In our experiment, *OPG* downregulation led to osteoclast differentiation and bone resorption, as detected by *TRAP* staining in the cortex. Interestingly, there was no difference in osteoclast activity at the chondro-osseous junction, highlighting the differences between osteoclasts in the GP and cortex. Correspondingly, we revealed increased porosity in the UPF group femoral cortex and similar TRAP mRNA levels in the UPF GP. In summary, the consumption of UPF stunted growth and disrupted bone quality and EO. The GP of the UPF group was occupied by a massive cartilaginous plaque that was assembled from a large number of cells and ECM. Transcriptome analysis revealed that this aberrant cartilage resulted from an imbalance in ECM formation and degradation, disrupted proliferation and differentiation mechanisms, and reduced mineralization.

Next, we wanted to understand what components of the UPD cause growth retardation and the bone phenotype. We demonstrated neither the soft drink nor the macronutrients was the cause, but the Ca and P deficiency and imbalance caused damage that was similar to but less severe than that caused by the UPD. This suggests that Ca/P deficiency leads to the aberrant phenotypes and that there are other cofactors responsible for these detrimental phenotypes in UPD.

One explanation might be the extensive processing of food during manufacturing. As established by Monterio et al. in the NOVA classification,^39^ oil or sugars may not be as detrimental to human health without additional or excess processing. Overprocessing itself is not always classified as a damaging process since well-balanced healthy products such as infant formulas are also included in this food group. However, the combination of sugar and oil overload with micronutrient deficiency and harmful processing procedures (such as partial hydrogenation of oils,^40^) may be the driving force behind impaired bone development. Behavioral changes in the rats consuming the UPF (extensive eating bordering on gluttony [Figs. 1d, 6d and 7a]) support this notion, since one of the results of ultraprocessing is the destructive chemical modifications needed to make the final product hyperpalatable.^39^

Finally, we established a UPD model in young rats that simulates modern Western eating habits. Providing rats with a whole diet that is equivalent to that eaten by humans is a novel scientific approach. The common nutritional methodology involves adding or removing one component at a time from the diet to study its effect separately, as in pharmaceutical or genetic studies. However, nutrition is different; a recent paper in Nature^41^ made the observation that “people do not choose nutrients, they select combinations of foods…researchers should be more creative and…bolder in assessing the health implications of common combinations of foods.” This is the research approach used here by studying the effect of a “whole diet” rather than the effect of individual nutrients, which in this case was the UPD or so-called “new malnutrition“.^42^ There is general agreement that the consumption of processed foods and sugar-sweetened beverages is associated with increased rates of obesity,^43^ diabetes^44^ and cardiovascular disease.^45,46^ We demonstrate a new target for the harmful effect of such a diet and suggest it as a direct risk factor for bone health.

## Material and methods

### Animal experiments

All experiments were conducted on female Sprague-Dawley (SD) rats after weaning (3 weeks of age). We chose young female rats before sexual maturation as our study model because female subjects are more prone to suffer from bone diseases. Thus, improving bone quality in young females is a preventative approach.

The rats were purchased from Harlan Laboratories (Rehovot, Israel) and housed under standard environmental conditions with a 12 h light:12 h dark cycle and *ad libitum* access to food and drink. After 4 days of acclimation, the rats were randomly divided into experimental groups. All procedures were approved by the Hebrew University Animal Care Committee #AG-11-13225-2, AG-13-13952-2. Throughout the experiments, body weight, body length from the tip of the nose to the end of the tail, food and fluid consumption were measured twice a week, and daily food intake in kilocalories was calculated for each rat per day. At two time points, after 3 and 6 weeks of the experiment (6 and 9 weeks of age, respectively), animals were anesthetized with isoflurane, blood samples were collected, and they were sacrificed. Their internal organs and bones (femur, tibia and spine) were harvested. The femora and 3^rd^–5^th^ lumbar vertebrae were manually cleaned of soft tissue and stored at −20 °C until micro-computed tomography (µCT) scanning and mechanical testing. Tibiae were fixed immediately after sacrifice (for histological studies) or frozen in liquid nitrogen for RNA sequencing analysis. For the full experimental setup of this study, see Supplementary Fig. 2.

### UPF experiment

Sixteen SD rats were divided into two groups. One group (*n* = 8) received a standard diet based on the composition for growing rats recommended by Harlan Laboratories (Control), and the other (*n* = 8) was offered a diet based on UPF, which was rich in fat and sucrose and included, a typical caloric soft drink (UPF + CSD). The UPD included a roll, hamburger, tomatoes, lettuce, ketchup (without onion or pickles) and French fries. The whole meal was homogenized, shaped into patties and frozen at −20 °C (Supplementary Fig. 2a).

### Macronutrient experiment

SD rats (*n* = 66) were divided into four groups that received (i) the standard diet for growing rats (Control, *n* = 16) and (ii) a diet based on UPF, which was rich in fat and sucrose and included a soft drink containing 10% sucrose (UPF + CSD, *n* = 18). In the third and fourth groups, the main ingredients of the fast food diet—fats and sugar—were isolated, and the animals received (iii) a high-fat diet with the addition of corn oil (Corn, *n* = 16) and (iv) a high-sucrose diet consisting of the regular control diet + drinking solution with 10% sucrose (sucrose, *n* = 16) (Supplementary Fig. 2b).

### Micronutrient experiment

To investigate whether the imbalance between calcium (Ca) and phosphorus (P) in the UPD causes the lesion phenotype in UPF-consuming rats, 24 rats were divided into three groups receiving (i) the standard diet (Control, *n* = 20); (ii) a diet based on UPF without the caloric soft drink (UPF, *n* = 20); and (iii) a custom diet with an altered Ca:P ratio, 62 mg Ca per 100 g and 121 mg P per 100 g of diet (0.06% and 0.12%, respectively) (*n* = 8). This ratio corresponded to the Ca:P ratio in the UPF group, while the other micronutrients and macronutrients were similar to those in the control diet. The diet was prepared by Envigo (Teklad laboratory animal diets; Madison, WI, USA) (Supplementary Fig. 2c).

### Eating pattern experiment

Twenty-four SD rats were divided into three groups that received (i) the standard diet (Control, *n* = 8); (ii) the UPD (UPF, *n* = 8); and (iii) the Control diet 30% of the week and the UPD the rest of the week (30%/70%, *n* = 8). Group (iii) mimicked UPF eating patterns (Supplementary Fig. 2d).

### Dietary composition

The macro- and micronutrient contents of the control and UPD diets were analyzed by Aminolab Laboratories (Rehovot, Israel), which provides analytical services certified by the FDA and Israeli Ministry of Health (Supplementary Fig. 5). This analysis was compatible with the information provided by Harlan Laboratories for the Control diet and the nutritional database of the US Department of Agriculture (www.ndb.nal.usda.gov/ndb/) for the UPD. Calories from each macronutrient were obtained by multiplying grams of protein, fats and carbohydrates by the factors 4-9-4, respectively.^47^ Supplementary Fig. 5 presents the caloric distribution of the macronutrients as percentages and the micronutrient contents of the different diets.

In Supplementary Table 4, we show the similarities between humans and rodents in the types of essential components required for daily functioning. These data emphasize the suitability of rats for nutritional studies.^48^

### Metabolic status analysis

Blood samples were collected for hematological and hormonal profile analyses. The complete blood count was performed at a veterinary teaching hospital (Bet Dagan, Israel), and hormonal profile analysis was performed at American Medical Laboratories (Herzliya, Israel).

### µCT analysis

Femora and lumbar vertebrae were scanned in a SkyScan 1174 X-ray computed microtomograph scanning device. Images were obtained with an X-ray tube voltage of 50 kV and current of 800 μA. The bones were scanned using a 0.25-mm aluminum filter with a 4 500 ms exposure time and at high spatial resolution (13.8 μm). For each specimen, a series of 900 projection images were obtained with a rotation step of 0.4° by averaging 2 frames for a total 360° rotation. For analysis of the diaphyseal cortical region, 200 slices were chosen. Global grayscale threshold levels for the cortical region between 87 and 255 were selected. For the trabecular analysis of the secondary ossification zone, 150 slices were selected, and the adaptive grayscale threshold levels of 85 and 255 were used.^49,50^

### Mechanical testing

The mechanical properties of the femora from each group were determined by three-point bending tests using a custom-made micromechanical testing device.^49^ The following bone biomechanical parameters were derived from load-displacement curves: bone stiffness, yield load, force to fracture, maximal force and total energy to fracture.^17,51,52^

### Histological analysis of the GP and bone sections

The effect of the different diets on the GP of growing rats was examined by histological techniques (to determine the structure and cell types). Histological staining included hematoxylin and eosin (H&E), safranin O, Masson’s trichrome staining, and tartrate-resistant acidic phosphatase (TRAP).^17,53^ In situ hybridization was performed to detect *Col2* and *Col10* expression.^54^ The widths of the whole GP and the PZ, HZ and lesion zone (LZ) were measured at 10 different points along the GP and averaged with measurements from 6 other plate samples in each group. The percentages of PZ and HZ/LZ from the whole GP were calculated. For the measurements, H&E staining was used. Stained tissue sections were viewed under an Eclipse E400 Nikon light microscope at various magnifications using light filters. Pictures were taken with an Olympus DP71 camera controlled by Cell A software (Olympus).

### Light microscopic images of cross sections of rat cortical bone

Transverse slices of ~1.2 mm were prepared from the middle of the diaphysis of the left femora from five rats. Each slice was marked to identify its axial position and proximal, medial and cranial aspects. The slices were ground and polished with successively finer abrasive paper (from 2 500 to 4 000 grit), followed by 3 and 1 cloth with diamond paste.^55^ The polished surfaces were studied using reflected-light microscopy (BX-51 microscope, Olympus, Japan). Images were captured using a 12.1-megapixel resolution DP71 digital camera attached to the microscope.

### Scanning electron microscopy (SEM)

Paraffin-embedded tibia bones were deparaffinized in xylene and rehydrated, carbon-coated and scanned. High-resolution images of the tibial GP surface were examined by SEM (JCM 6000, Jeol, Tokyo, Japan) at various working distances with an electron energy of 10 keV in high-vacuum mode using secondary electron detector mode.

### RNA extraction, mRNA library preparation and sequencing

After 3 weeks of the experiment, animals were sacrificed, and the right tibia bones from the control (*n* = 8) and UPF (*n* = 8) groups were collected. The GP area was immediately isolated with a scalpel, avoiding contamination with adjacent unwanted tissue (such as bone, articular cartilage, bone marrow, muscle ligament and tendon). The isolated GP was then pulverized manually in liquid nitrogen. Total RNA was extracted using TRI reagent (Sigma, St. Louis, MO, USA) according to the manufacturer’s protocol. The structural integrity of the RNA is of great importance in preparing mRNA libraries. Therefore, we used the Agilent 2200 TapeStation system to assess RNA quality. The TapeStation instrument produces gel images and RNA integrity numbers (RIN) for each sample. The RIN is presented as a value between 1 and 10, where 10 represents the highest quality RNA.^56^ Twelve samples with high RIN values (>7) were selected for mRNA library preparation. Nine libraries (control *n* = 4 and UPF *n* = 5) were successfully prepared using Lexogen’s QuantSeq 3’ mRNA-Seq library prep kit. Libraries were quantified by a Thermo-Fisher Qubit and Agilent TapeStation and sequenced in an Illumina NextSeq 500 sequencer using the NextSeq 75 bp kit to produce single-end reads. The output was ~25 million reads per sample. The sequencing process was conducted by the Core Research Facility at The Faculty of Medicine - Ein Kerem, The Hebrew University of Jerusalem, Israel.

### Bioinformatics

Raw reads (fastq files) were inspected for quality with FastQC v0.11.4. Raw reads were trimmed for quality to remove the poly (A) tails and adapter sequences using the Trim Galore default settings.

Single-end reads were mapped to the rat genome (Rattus_norvegicus. Rnor_6.0.94) using STAR v2.201. Mapping files were further processed for visualization by Samtools Utilities v 0.1.19.

Differential expression analysis: The uniquely mapped reads per gene were counted using HTSeq-count, and differential expression analysis was performed using the DESeq2 R package. Genes were considered significantly expressed if the adjusted *P* value was lower than 0.05. Additionally, we used a fold change filtering value of |fold change | >1.

GeneAnalytic was used to search for possible biological processes, canonical pathways and networks. This software is a gene set analysis tool for contextualization of expression patterns and functional signatures embedded in postgenomics big data domains, such as RNA sequences. To perform this analysis, we converted the rat gene IDs to those of the homologous human genes using the OMA web-based database interface for orthology prediction. Next, using Biomart, we converted the human IDs to NCBI IDs. We then uploaded the differentially expressed genes to the GeneAnalytics website.^18^

### Statistical analysis

All data are expressed as the mean ± SD (standard deviation). The significance of differences between groups was determined using JMP 12.0 Statistical Discovery Software (SAS Institute 2000) by one-way ANOVA followed by the Tukey–Kramer HSD test and t-test. Differences were considered significant at *P* ≤ 0.05. Groups with the same letter were not measurably different, and groups that were measurably different are indicated by different letters. Groups may have more than one letter to reflect the “overlap” between the sets of groups, and sometimes a set of groups is associated with only a single treatment level.

## Supplementary information

Supplemented material
