# Supplementary material for: Ultra-processed food targets bone quality via endochondral ossification
Source: Bone Res. 2021 Feb 26;9:14. doi: 10.1038/s41413-020-00127-9 (PMC7910299; doi:10.1038/s41413-020-00127-9)
Supplement: Supplementary file 1 — Supplemented material [file 41413_2020_127_MOESM1_ESM.docx]

**Supplementary Table 1.**

**Consumption of ultra-processed diet leads to alterations in bone architecture and biomechanical properties.** Femora and vertebrae of 6- and 9-week-old rats were subjected to µCT scan. Trabecular bone parameters: bone volume fraction (BV/TV), trabecular number (Tb.N), trabecular separation (Tb.Sp) and trabecular thickness (Tb.Th). Cortical bone parameters: cortical area fraction (Ct.Ar/Tt.Ar), average cortical thickness (Ct.Th), medullary area (Ma.Ar), bone mineral density (BMD), percentage of object volume (Obj.V/TV), cortical porosity (Ct.Po), pore number (Po.N) and total pore volume (Po.V). Three-point bending was used to measure the biomechanical properties of femora, derived from load-displacement curves. Values are expressed as mean ± SD, n = 8. Different letters denote significant difference at *P* < 0.05 between groups. See legend to Supplementary Figure 1 for description of different dietary groups.

**Supplementary Table 2.**

Log 2-fold change of differentially expressed genes (p<0.05) clustered according to extracellular matrix (extracellular matrix, collagen chain trimerization, Articular cartilage ECM, ECM protein receptor interaction and endochondral ossification) and general pathways (Integrin, PAK, ERK, phospholipase-C, neuroscience and adipogenesis).

**Supplementary Table 3. Serum analysis.**

(a) Hormonal profile and (b) serum analysis of rats from the macronutrient experiment. (c) Hormonal profile and (d) serum analysis of rats from the micronutrient experiment. (e) Serum analysis of rats from the eating-pattern experiment. Values are expressed as mean ± SD. Different letters denote significant difference at P < 0.05 between groups. See legend to Supplementary Figure 1 for description of different dietary groups.

**
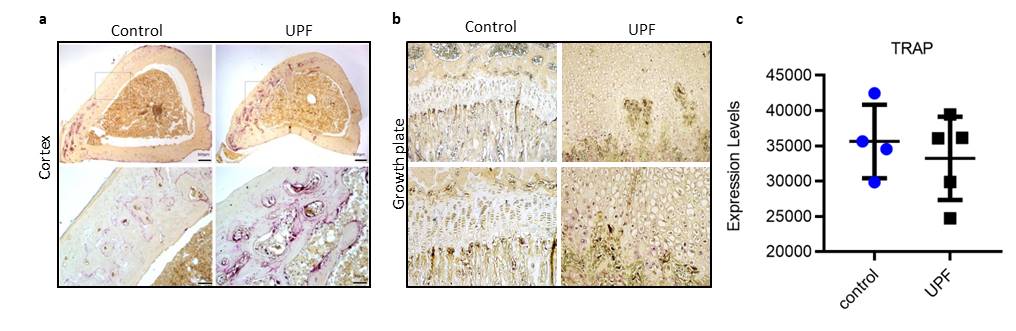
**

**Supplementary Figure 1.** Tibiae from the Control and UPF+CSD groups were dissected, processed, embedded in paraffin blocks and subjected to TRAP staining. (a) Cortical bone. (b) Growth plate (GP). (c) Expression frequency of TRAP gene in the GP.


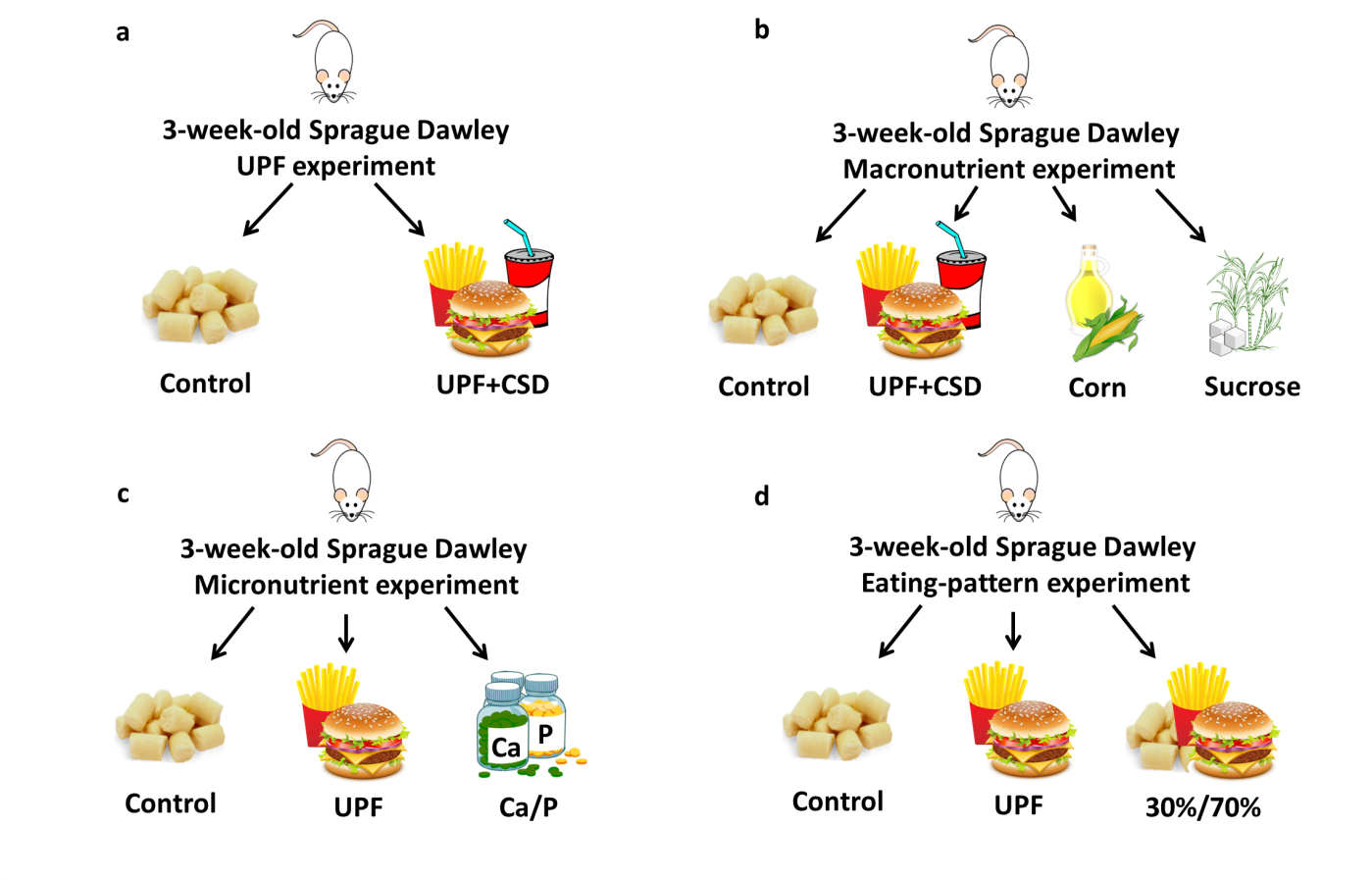


**Supplementary Figure 2. Schematic illustration of the experimental setups.**

(a) **UPF experiment:** The Control group (n = 8) received a standard diet based on the Harlan Laboratories recommended composition for growing rats; the UPF+CSD group (n = 8) received a diet rich in fat and sucrose, composed of a homogenized McDonald’s meal and a soft drink.

(b) **Macronutrient experiment**: The Control (n = 16) and UPF+CSD groups (n = 18) were compared to the Corn group (n = 16), which received a high-fat diet based on the addition of corn oil; and the Sucrose group (n = 16), which received a high-sucrose diet based on the Control diet + drinking solution with 10% sucrose.

(c) **Micronutrient experiment:** The Control group received a standard diet (n = 20), the UPF group (n = 20) received a diet based on UPF without the caloric soft drink (UPF), and the Ca/P group (n = 8) received a custom diet with altered Ca to P ratio: 62 mg Ca per 100 g and 121 mg P per 100 g diet (0.06% and 0.12%, respectively). This ratio corresponded to the Ca/P ratio in the UPF group, while the other micronutrients and macronutrients were as in the Control diet.

(d) **Eating-pattern experiment:** The Control group (n = 8) and the UPF group (n = 8) were compared to the 30%/70% group (n = 8) which received a Control diet 30% of the week and the UPF diet the rest of the week (30%/70% group)

**Supplementary text**

**Caloric and non-caloric soft drinks do not affect** **growth, physiological parameters and bone quality.**

To study the effect of caloric and non-caloric soft drinks (Coca-Cola or Coca-Cola Zero) on growth, physiological parameters and skeletal development, an *in-vivo* experiment was conducted on young female rats for a duration of 6 weeks until puberty. The experimental setup included 48 Sprague Dawley rats divided into three groups, receiving: (i) the standard diet for growing rats (Control, n = 16); (ii) a regular control diet and caloric soft drink (CSD group, n = 16); (iii) a regular control diet and a non-caloric soft drink (NCSD group, n = 16). The rats received *ad libitum* access to food and water along with access to the soft drink.

During the experiment, body weight and total body length (from the tip of the nose to the end of the tail) were measured. At day 25, the CSD consuming rats, had higher body weight compared with the NCSD group but did not differ from the Control group (Supplementary Fig. 3a). Total body length was measured in three time points during the experiment. In the second measurement, the length of the CSD group was lower compared with the other groups. However, by the end of the experiment, this group was longer compared with the Control group but did not differ from the NCSD group. In spite of the minor differences in body length, femora length was similar between the groups (Supplementary Fig. 3b-c). Total drinking volume showed that addition of soft drink (CSD or NCSD) caused a decrease in water consumption. The percentage of soft drink volume intake was 54% and 57% in the CSD and NCSD groups, respectively. Total drinking in the CSD group was significantly higher compared with the Control and NCSD groups (Supplementary Fig. 3d). The CSD group consumed significantly more calories, despite the fact that caloric soft drink partially replaced their food intake (Supplementary Fig. 3e-f).

Analysis of the trabecular properties of the femora revealed that only Tb.Th was significantly lower in the CSD group compared with the control (Supplementary Fig. 3g). Analysis of the cortical bone properties showed no difference between the diets. Mechanical bone properties, which were analyzed by three point bending test, revealed no differences between the three groups consistent with the mild differences in their morphology. Histological examination of the tibiae bones showed normal structure and organization of the GP in all three groups (Supplementary Fig. 3h).

Altogether, these results demonstrate that consumption of solely soft drinks alone does not seriously affect growth, bone quality or structure.

**Consumption of ultra-processed diet with or without caloric soft drinks (CSD) leads to growth retardation and to alteration in bone architecture and biomechanical properties.**

To further study the effect of soft drink on metabolic and bone parameters another animal experiment was conducted. The experimental setup included 24 Sprague Dawley rats that were divided into three groups, receiving: (i) the standard diet for growing rats (Control, n = 8); (ii) a diet based on UPF, rich in fat and sucrose and including a soft drink containing 10% sucrose (UPF+CSD, n = 8); (iii) a diet based on UPF without the caloric soft drink (UPF, n = 8).

UPF + CSD group consumed more calories compare to UPF group, as can be seen in daily eating patterns graph (Supplementary Fig 4a). UPF + CSD and UPF groups exhibited similar stunted growth patterns in weight, body length and femora length (Supplementary Fig. 4b–d).

Trabecular and cortical bone parameters did not differ between UPF + CSD and UPF group (Supplementary Fig. 4e-l). Moreover, bone mechanical parameters were similarly diminutive in UPF + CSD and UPF groups (Supplementary Fig 4m).

GP organization was altered in UPF + CSD and UPF groups. Both of the group presented a mass of avascular non-mineralized cartilage plaque in their GPs (Supplementary Fig 4n). UPF + CSD and UPF groups demonstrated a variety of lesions (size, shape and localization). Measurement of the GP revealed similar disproportion in the different GP regions in UPF + CSD and UPF groups (Supplementary Fig 4o).

Taking together, no dramatic differences were found between the rats consuming UPF with or without soft drink in terms of metabolic parameters, or bone quality, structure or growth. Hence, we concluded that the harmful effects were not caused solely by the soft drink.


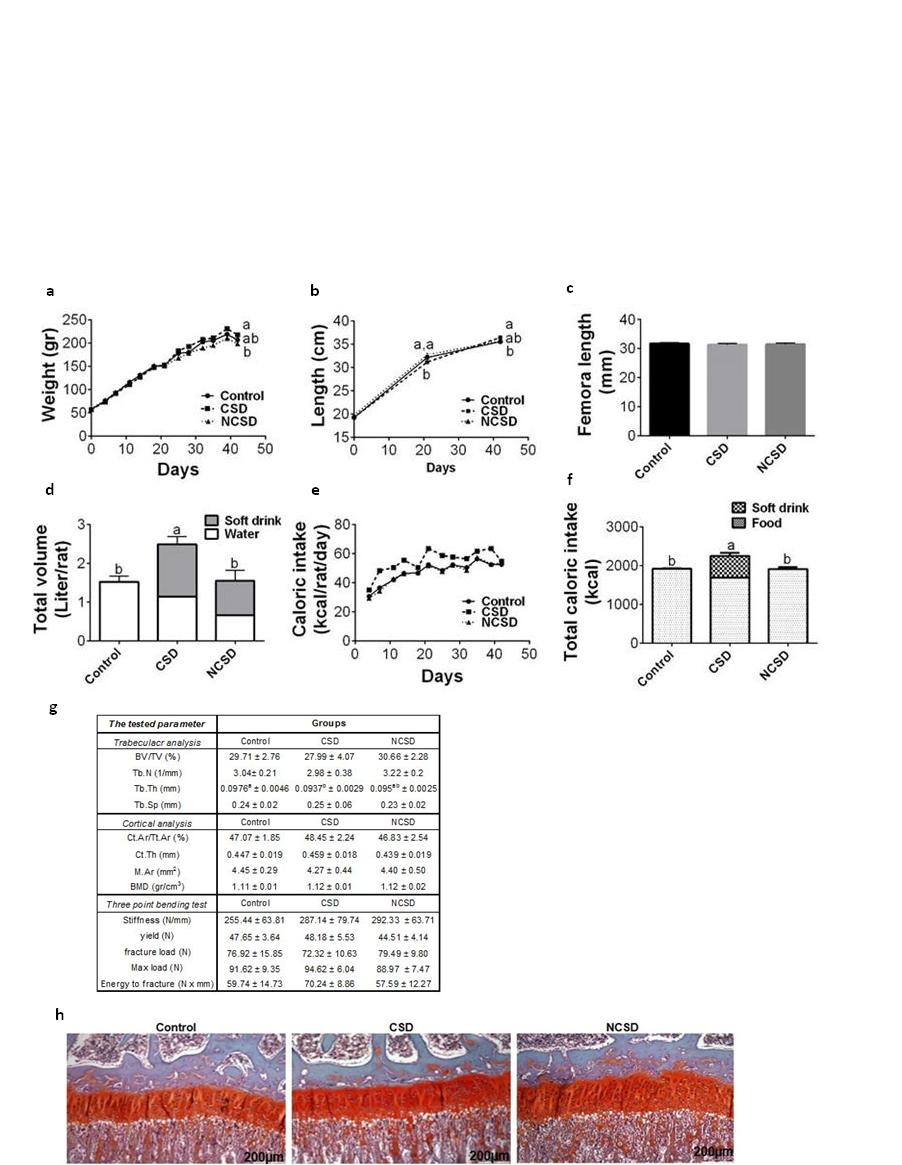


**Supplementary Fig. 3**

**Effect of caloric soft drinks (CSD) and non-caloric soft drinks (NCSD) on physiological parameters and bone development.** The Control group, which received a standard diet for growing rats, was compared to a group that received a control diet and CSD and to a group that received control diet and NCSD. (a) Body weight. (b) Total length from nose to tail, at 3 time points. (c) Femora length in 9 weeks old rats. (d) Total fluid (water and soft drink) consumption for the entire experiment. (e) Daily caloric intake (Kcal/rat/day) and (f) total caloric consumption for the entire experiment. (g) Femora of 9 weeks old rats were subjected to µCT scan. Trabecular bone parameters: bone volume fraction (BV/TV), trabecular number (Tb.N), trabecular thickness (Tb.Th) and trabecular separation (Tb.Sp). Cortical bone parameters: cortical area fraction (Ct.Ar/Tt.Ar), average cortical thickness (Ct.Th), medullary area (Ma.Ar), bone mineral density (BMD). Three-point bending was used to measure the biomechanical properties of femora, with parameters derived from load-displacement curves. (h) Tibiae were dissected, processed and stained for safranin-O to assess cartilaginous GP morphology. Values are expressed as mean±SD, n=8. Different letters denote p<0.05 between groups.


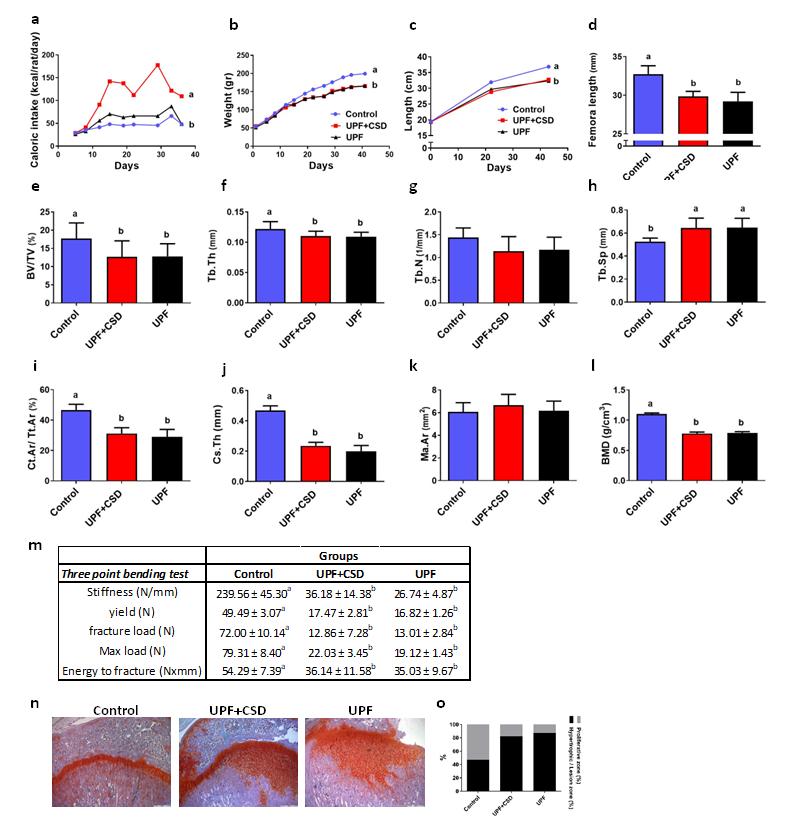


**Supplementary Fig. 4**

**Consumption of ultra-processed diet with or without caloric soft drinks (CSD) leads to growth retardation and to alteration in bone architecture and biomechanical properties.** The Control group (n=8) and the UPF+CSD group (n=8) were compared to UPF group that received McDonald’s meal without the soft drink. (a) Daily caloric intake (Kcal/rat/day). (b) Body weight. (c) Total length from nose to tail. (d) Femora length at 9 weeks of age. (e) Vertebrae length. (e-l) Femur µCT analyses. (e-h) Trabecular parameters: bone volume fraction (BV/TV), trabecular number (Tb.N), trabecular thickness (Tb.Th), and trabecular separation (Tb.Sp). (i-l) Cortical parameters: cortical area fraction (Ct.Ar/Tt.Ar), average cortical thickness (Ct.Th), medullary area (Ma.Ar) and bone mineral density (BMD). (m) Biomechanical properties: stiffness (N/mm), yield (N), fracture load (N), max load (N) and energy to fracture (N x mm), assessed by three-point bending test. (n) Tibiae from Control, UPF + CSD and UPF groups were stained dissected, processed, embedded in paraffin blocks and stained with safranin-O. (o) Quantification of the relative ratio of the zones in the GP. Values are expressed as mean ± SD, n = 8. Different letters denote p<0.05 between groups.

**
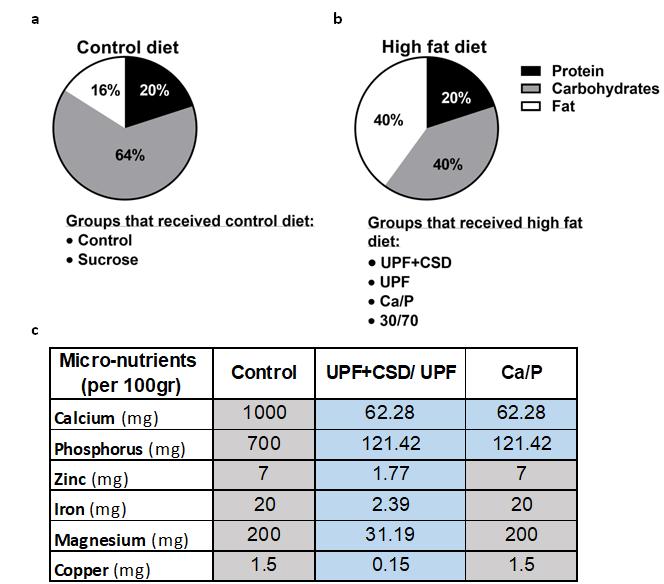
**

**Supplementary Fig. 5**

**Schematic illustration of diet composition.** (a) Caloric distribution of macronutrients in the Control diet (received by Control and Sucrose groups). (b) Caloric distribution of macronutrients in the high-fat diet (received by UPF an UPF+CSD groups). (c) Micronutrient composition of the different diets. See legend to Supplementary Figure 1 for description of different dietary groups.

**Supplementary Table 4.**

**Essential dietary components in humans and rats.** Similarities between humans and rodents with respect to essential components required for daily function*.*
